# Institutional Needs

**Published:** 1914-01-03

**Authors:** 


### Institutional Needs.

### AN ENGINEERING CATALOGUE.

Manlove, Alliott and Co., Ltd., of Bloomsgrove Works, Nottingham, have issued a very instructive circular epitomising their hospital engineering products, which is well worth consulting by managers or committees considering the installation of any new plant. Their steam cooking apparatus, including wet steam ovens, hot closets and carving tables, air heaters, centrifugal machines, steam disinfectors, and laundry machinery, are capitally illustrated and epitomised. Circular 473 B also illustrates the valuable steam sterilisers made by the firm, the oil and gas heated, the rotary type, and the steam and gas heated all being shown. In addition to the manufacture of complete individual machines, accessories of every class, including valves, gauges, pipes, bends, tees, and coils, are also obtainable. Inquirers should give as much specific information as possible, since it is possible here merely to indicate the type of machinery which is supplied by the firm. Its London offices are at 41 and 42 Parliament Street, Westminster, while there is also a branch at 23 King Street, Manchester. Every hospital engineer should be furnished with a copy of the above circular.

### SOFT CORK CUSHIONS.

Lioline Edwards, 81 St. Margaret's Road, Twickenham, have placed on the market what is known as the "Quidos" soft cork cushion seat. Filled with granulated cork, the cushions are made to fit any ordinary chair, and are covered with green rot-proof canvas. The object of the cushions is to provide air cells of twice the ordinary size, so as to secure resiliency, non-conductivity of heat, and hard wear. The cushions are of two types, square with the canvas sewn in parallel divisions, and a round one with four compartments of cork, and a centre which is hollow except for the canvas sewn across it. The makers believe the cushion to be absolutely sanitary, as no germs can find harbourage in it. The price of either pattern is 2s. 6d., and the cushions are extremely comfortable and likely to be useful either in an office or while travelling.

### ACITRIN.

[The Bayer Co., Ltd.]

The pathogeny of gout still remains one of the speculative departments of modern medicine, and usually inquirers are put off with the statement that gout is due to faulty metabolism. But the importance in gouty conditions of promoting the excretion of uric acid is recognised on all sides, and therefore any means by which this can be efficiently accomplished without detriment are deserving of consideration. As serving one at least of the indications of medical treatment the new drug introduced by the Bayer Co., Ltd., under the name of "Acitrin" is likely to find favour among practitioners. Chemically it is an ethyl ester of phenyl cinchonin acid, and it is insoluble in water but tasteless. Experiments in a large number of cases have shown that the excretion of uric acid is materially increased after the administration of a few doses of acitrin. This drug is obtainable in tablets weighing 7½ grains, which are sold in tubes containing twenty. Trial supplies of the preparation will be sent to any medical man interested.

### THE WELLCOME DIARY.

The Pocket Diary for the record rather of things to be done than of things perhaps better left undone is essential to everyone with engagements to keep, and the one produced by Burroughs Wellcome and Co. is a well-established favourite. It includes also a photographic exposure record, with every hint that experience can suggest to give the amateur the certainty and precision of the expert photographer. Exposures can be calculated systematically by means of the "record," and the combined Diary and Record makes a capital New Year present, which is obtainable at all bookstalls for 1s.

